# Predominance of *Giardia duodenalis* AII sub-assemblage in young children from Salvador, Bahia, Brazil

**DOI:** 10.7705/biomedica.5161

**Published:** 2020-06-30

**Authors:** Flávia Thamiris Figueiredo Pacheco, Renata Kelly Novaes Rodrigues Silva, Silvia Souza de Carvalho, Felipe Carvalho Rocha, Gisele Maria Trindade das Chagas, Daisy Chagas Gomes, Hugo da, Tereza Cristina Medrado Ribeiro, Ângela Peixoto de Mattos, Luciano Kalabric Silva, Neci Matos Soares, Márcia Cristina Aquino Teixeira

**Affiliations:** 1 Faculdade de Farmácia, Universidade Federal da Bahia, Salvador, Brasil Universidade Federal da Bahia Universidade Federal da Bahia Salvador Brazil; 2 Centro Pediátrico Professor Hosannah de Oliveira, Universidade Federal da Bahia, Salvador, Brasil Universidade Federal da Bahia Centro Pediátrico Professor Hosannah de Oliveira Universidade Federal da Bahia Salvador Brazil; 3 Fundação o Oswaldo Cruz, Centro de Pesquisa Gonçalo Moniz, Salvador, Brasil Fundação o Oswaldo Cruz Centro de Pesquisa Gonçalo Moniz Salvador Brasil

**Keywords:** Giardiasis/epidemiology, child, daycare centers, Brazil, giardiasis/epidemiología, niño, guarderías, Brasil

## Abstract

**Introduction.:**

*Giardia duodenalis* is an intestinal protozoan with a high prevalence in children of developing countries. Molecular studies revealed a great genetic diversity of *G. duodenalis*, with assemblages A and B found mainly in humans. Despite its importance, the information on the molecular epidemiology of human giardiasis is still limited in Brazil.

**Objective.:**

To characterize *G. duodenalis* molecular isolates in children from Salvador, Bahia, Brazil.

**Materials and methods.:**

*Giardia duodenalis* positive fecal samples were obtained from 71 children from two day care centers and 39 users of a clinical analysis laboratory. Samples were analyzed by PCR-RFLP of the glutamate dehydrogenase (*gdh*) and *beta-giardin* genes and by the sequencing of *beta-giardin*.

**Results.:**

Of the 110 *G. duodenalis* samples, 80 (72.7%) amplified one or both target genes. Of these, 62 (77.5 %) were identified as assemblage A and 18 (22.5%) as assemblage B. The subassemblage AII was identified in 58.8% (n=47) of isolates followed by the sub-assemblage AI (18.8%, n=15), BIV (11.2%, n=9), and BIII (5.0%, n=4). The AII sub-assemblage was the most frequent in children of both day care centers whereas AI was found only in the group attended at the clinical laboratory. Sub-assemblage AII predominated in children under two years.

**Conclusions.:**

The higher frequency of AII sub-assemblage suggests that anthroponotic transmission is more common in Salvador, but that zoonotic transmission pathways are also present and a change in susceptibility to different molecular patterns of *Giardia* may occur during child growth.

## Resumen

**Introducción.:**

*Giardia duodenalis* es un protozoo intestinal de gran prevalencia en los niños de los países en desarrollo. En estudios moleculares se ha evidenciado la gran diversidad genética de *G. duodenalis* y se han identificado los conjuntos A y B, principalmente en humanos. A pesar de su importancia, el conocimiento de la epidemiología molecular de la giardiasis humana aún es limitado en Brasil.

**Objetivo.:**

Caracterizar los aislamientos moleculares de *G. duodenalis* de muestras tomadas a niños de Salvador, Bahía, Brasil.

**Materiales y métodos.:**

Las muestras fecales positivas para *G. duodenalis* se obtuvieron de 71 niños de dos guarderías y de 39 usuarios de un laboratorio de análisis clínicos. Las muestras se analizaron mediante PCR-RFLP de los genes *gdh* y *beta-giardin*, y secuenciación de *beta-giardin*.

**Resultados.:**

De las 110 muestras de *G. duodenalis*, en 80 (72,7 %) se amplificaron uno o ambos genes. De estos, 62 (77,5 %) se identificaron como pertenecientes al conjunto A y 18 (22,5 %) al B. El subconjunto AII se identificó en el 58,8 % (n=47) de los aislamientos, seguido del AI en el 18,8% (n=15), el BIV en el 11,2% (n=9) y el BIII en el 5,0% (n=4). El subconjunto AII fue el más frecuente en los niños de ambas guarderías, en tanto que el AI solo se encontró en el grupo atendido en el laboratorio clínico. El subconjunto AII predominó en los niños menores de dos años.

**Conclusiones.:**

La mayor frecuencia del subconjunto AII sugiere que la transmisión antroponótica es más común en Salvador, pero también existen vías de transmisión zoonóticas, y que pueden ocurrir cambios en la sensibilidad frente a diferentes patrones moleculares de *Giardia* durante el crecimiento infantil.

Giardiasis is of considerable public health importance in developing countries due to its high prevalence in young children and its effects on early childhood diarrhea and malnutrition ^1^^-^^3^. The high susceptibility of children to *G. duodenalis* infection is usually attributed to the immaturity of their immune system when the first contact with the parasite occurs and poor hygiene habits compared with those of adults ^1^. The transmission of giardiasis occurs via the fecal-oral route and infection results from the ingestion of cysts present in food or water contaminated with feces ^4^^,^^5^. Direct transmission from person to person also contributes to the dissemination of the parasite among children attending day care centers and schools ^1^^,^^6^.

Although *G. duodenalis* is considered a unique species, advances in molecular biology techniques have revealed that the protozoan is a complex of species with genetic diversity but morphologically identical, which exhibits adaptation to different hosts ^4^^,^^7^^,^^8^. The related *Giardia* genotypes have been grouped into the eight main assemblages, A, B, C, D, E, F, G, and H, and their respective sub-assemblages ^4^^,^^9^^,^^10^. Differences in the gene sequences coding assemblages A and B have made it possible to distinguish genetic groups and subgroups which differ in host specificity ^11^. Assemblage A was classified into sub-assemblages AI to AIV where AI is usually reported in humans and animals, AII is exclusive to man, and AIII and AIV are unique to animals ^12^. Assemblage B includes sub-assemblages III and IV identified in fecal samples obtained from humans, dogs, cats, horses, calves, and wild animals ^11^^,^^13^.

The geographical distribution of *G. duodenalis* human assemblages varies greatly around the world. In countries such as Bangladesh ^14^, Portugal ^15^, Germany ^16^, Uganda ^17^, and Syria ^18^, studies have reported the predominance of assemblage A*.* However, a higher prevalence of human infections by assemblage B was observed in Austria (19, Kenya ^20^, Libya ^21^, Canada ^22^, Egypt ^23^, and Argentina ^24^.

In Brazil, there are few studies describing the distribution *G. duodenalis* genotypes in humans. In Rio de Janeiro, Volotao, *et al.*^25^, identified only assemblage A, mostly classified as AII. In Sao Paulo, the analysis of five isolates of axenic trophozoites had the same results as in Rio, i.e., only assemblage A, mostly AII ^26^. However, in another study conducted in Sao Paulo with isolates from children in day care centers, assemblage B predominated ^27^ while in Fortaleza, Kohli, *et al*. ^28^, amplified 58 isolates and found assemblage B in 74.1% of them, A in 15.5%, and mixed infections (A + B) in 10.3% whereas in the state of Minas Gerais, only type B was found ^29^. Recently, assemblage B was also reported in patients from the metropolitan area of Rio de Janeiro evidencing changes in the frequency patterns of assemblages A and B over the five-year study ^30^.

Notwithstanding the high frequency of *G. duodenalis* infection in Brazil, mainly in young children, the molecular epidemiology of the parasite has been poorly studied, especially in the northeastern region. In the present study, we characterized *G. duodenalis* isolates from preschool and schoolchildren in Salvador, Bahia, Brazil.

## Materials and methods 

### Origin of samples

*Giardia*-positive stool samples were obtained from children up to 6 years old from two day care centers (46 from day care center 1 and 25 from day care center 2) supported by philanthropic institutions and from 39 children under 14 years of age attending the clinical analysis laboratory of the Faculty of Pharmacy at the Federal University of Bahia. All children were users of health public services and came from low-income families. Positive samples were identified by centrifugalsedimentation in water ^31^, centrifugal-fluctuation in zinc sulfate ^32^, and/or by coproantigen detection using a specific commercial enzyme immunoassay (ELISA; RIDASCREEN *Giardia* ^™^, R-Biopharm AG, Germany). To compare the frequencies of specific protozoa assemblages and sub-assemblages, children infected with *G. duodenalis* were divided according to their age and gender.

### Molecular characterization of G. duodenalis

*DNA extraction from feces and PCR conditions.* DNA from *G. duodenalis* cysts was purified using QIAamp DNA Stool Mini Kit^™^ (Qiagen, Hilden, Germany) following the manufacturer's instructions with some modifications. For example, the time and temperature of the cell lysis step were increased to 10 min at 95°C and the DNA elution volume was reduced to 100 μl of the buffer.

A 753-bp fragment of the *beta-giardin* gene was amplified using forward primer G7 and reverse primer G759 ^7^. In the sequential nested PCR reaction, a 511-bp fragment was amplified using forward primer G99 and reverse primer G609 ^33^. In all cases, the PCR mixture consisted of 1X buffer containing 1.5 mM MgCl_2_, 200 μl of each dNTP, 10 pmol of each primer, 2.5 units of *Taq* DNA polymerase (Invitrogen), and 1 μl of purified DNA in a final volume of 25 μl. The PCR reactions were performed as follows: An initial denaturation step of 5 min at 94°C for the first PCR and 15 min at 95°C for the nested-PCR followed by 35 cycles of 30 sec at 94°C, 30 sec of annealing (65°C for the primary *beta-giardin* PCR and 55°C for the nested PCR), and 60 s at 70°C with a final extension of 7 min at 72°C.

Additionally, *G. duodenalis* isolates identified as genotype A through the analysis of the *beta-giardin* gene were subjected to a semi-nested PCR (sn- PCR) for amplification of the 384-bp fragment using the direct primers G376 and reverse G759 under the same PCR conditions used for the amplification of the 753-bp *beta-giardin* fragment ^7^.

A 432-bp fragment of the *gdh* gene was amplified using semi-nested PCR as previously described ^34^. In the primary PCR reaction, the DNA fragment was amplified using forward primer *GDH*eF and reverse primer *GDH*iR. In the sequential semi-nested PCR reaction, a 432-bp fragment was amplified using forward primer *GDH*iF and reverse primer *GDH*iR. In all cases, the PCR mixture consisted of 1X buffer containing 2 mM MgCl_2_, 200 μM of each dNTP (GC:TA = 3:1), 12.5 pmol of each primer, 1 unit of *Taq* DNA polymerase (Invitrogen), and 1 μl of purified DNA in a final volume of 25 μl for the primary PCR and 50 μl for the sn-PCR. The PCR reactions were performed as follows: An initial denaturation step of 5 min at 94°C followed by 40 cycles consisting of 30 s at 94°C, 20 s of annealing at 65°C and 45 s at 72°C with a final extension of 7 min at 72°C. All PCR products were analyzed by electrophoresis on ethidium bromide-stained 1% agarose gels.

*Amplicon analyses by RFLP and sequencing.* For the characterization of *Giardia* assemblages, 10 μl of the 511 bp *beta-giardin* amplicon were digested overnight with 10 U of *Hae*III in a final reaction volume of 32 μl at 37°C (7). For identification of A sub-assemblages (AI, AII/AIII), the 384 bp fragment produced by snPCR was digested with the endonuclease *Hha*I as described above ^33^. The *gdh* gene was digested overnight at 37°C using 10 μl of the 432 bp amplicon of the snPCR and 10 U of the enzyme *Nla*IV (*BspL*I) in a final volume of 32 μl. Samples indicating the presence of assemblage B had the amplicons also digested with a second endonuclease, the *Rsa*I, under the same conditions to specify sub-assemblages BIII and BIV ^34^. Restriction fragments were analyzed by 3% agarose gel electrophoresis using a 50 bp molecular weight standard. The electrophoresis run was performed at 100 volts for two hours.

The isolates with mixed genotype patterns or inconclusive RFLP results were submitted to amplicon sequencing of the *beta-giardin* gene. PCR products were purified and sequenced by the Macrogen Inc. sequencing service (Macrogen Inc., Seoul, Korea). Nucleotide sequences and electropherograms were analyzed and edited using the program CLC Main Workbench^™^, version 8.0 (CLC Bio, Qiagen). To determine the genotype of each sample, the tree phylogenetic analysis was performed using the neighbor-joining method using the MEGA 6 software ^35^. *Beta-giardin* gene references corresponding to the different *G. duodenalis* assemblages or sub-assemblages were obtained from GenBank (AY072723, sub-assemblage AII; KR051224, sub-assemblage AI; GQ337974, assemblage B; AY072726, sub-assemblage BIII; AY072725, subassemblage BIV; and GQ337973, assemblage E). Sequences were deposited in GenBank under accession numbers MG845536 to MG845549.

### Statistical analysis

The data were analyzed using the IBM SPSS^™^ software for Windows and the statistical analyses were performed with the GraphPad Instat^™^ program (GraphPad Software, Inc., San Diego, California, USA). The chi-squared test was used to compare the frequency of *G. duodenalis* assemblages and subassemblages according to the age and gender of children while the Kruskal- Wallis followed by Dunn post-test was performed to compare numerical variables. A probability of less than 0.05 was considered significant.

### Ethical considerations

The Ethics Committee of the Nursing School at the Federal University of Bahia, Brazil, approved the study (project approval number 907.867).

Children whose parents agreed to participate in the study and signed an informed consent form were enrolled during the research period. Children over 8 years of age were also informed about the research and signed a consent form. All parasitological test results were sent to the children's parents and individuals with parasitic infections were adequately treated by pediatricians when necessary. 

## Results

### Genotyping and subgenotyping of G. duodenalis isolates

From the 110 samples positive for *G. duodenalis*, 80 (72.7%) had the DNA successfully amplified in one or both genes (Table 1). Fifty-three (48.2%) isolates were amplified in both loci analyzed, 6 (5.4%) amplified only *beta- giardin,* and 21 (19.1%) only *gdh* (Table 1).

**Table 1:** Frequency of *beta-giardin* and *gdh* genes amplification

Target gene	n (%)
*beta-giardin*	6 (5.4)
*gdh*	21 (19.1)
*beta-giardin* + *gdh*	53 (48.2)
Non-amplified	30 (27.3)
Total	110 (100.0)

The PCR-RFLP analysis of both target genes and the sequencing of *beta- giardin* revealed assemblage A as the most frequent in the general population as it was found in 77.5% (62/80) of the isolates (p<0.05). Assemblage B was identified in 22.5% (18/80) of the *G. duodenalis* samples (Table 2).

**Table 2:** Distribution of *Giardia duodenalis* assemblages by children groups

Frequency of assemblages and sub-assemblages in children groups				
n (%)				
	Day care center 1 (n=31)	Day care center 2 (n=24)	Laboratory users (n=25)	Total (n=80)
Assemblages				
A	26 (83.9)^a,b^	13 (54.2)	23 (92)^c^	62 (77.5)^d^
B	5 (16.1)^a,e^	11 (45.8)^e^	2 (8)^e^	18 (22.5)^d^
Sub-assemblages				
AI	-	-	15 (60.0)	15 (18.8)^c^
AII	26 (83.9)^a,b^	13 (54.2)^b^	8 (32.0)^b^	47 (58.8)^c,d,e^
B (non-subtyped)	1 (3.2)	3 (12.5)	1 (4.0)	5 (6.2)
BIII	4 (12.9)^a^	-	-	4 (5.0)^d^
BIV	-	8 (33.3)	1 (4.0)	9 (11.2)^e^

^a^ ^bcde^ Equal letters indicate statistically significant differences (p<0.05, chi-squared test) in the frequency of assemblages and sub-assemblages among the groups.

When groups were analyzed separately, assemblage A was significantly more frequent than B (p<0.05) in samples from day care center 1 and laboratory users, whereas in day care center 2 there was no statistical difference in the occurrence of these two genetic types (Table 2). Assemblage B in children was significantly more frequent in day care center 2 (11/18, 61.1%, p<0.05) than in the other groups.

Overall, sub-assemblage AII was the most frequent (47/80, 58.8%) followed by AI (15/80, 18.8%). Of the 18 *G. duodenalis* samples identified as assemblage B, 13 were successfully sub-classified as BIII (5.0%) and BIV (11.2%) (Table 2).

*Giardia duodenalis* sub-assemblage distribution also differed among groups: AI was found only in children's samples from the routine laboratory and 10 of these 15 isolates (66.7%) were from children under six years of age, i.e., in the same age range as children from the day care centers. A significant predominance of AII sub-assemblage (p<0.05; 83.9%) was observed in day care center 1. On the other hand, although AII was the most frequent (54.2%) type in day care center 2, no significant difference was found compared to assemblage B frequency (45.8%). In both day care centers, only AII was detected among *G. duodenalis* isolates identified as assemblage A.

### Distribution of sub-assemblages by gender and age

There was no significant difference in *G. duodenalis* sub-assemblage the distribution as regards children's gender but there was a difference regarding their age: AI sub-assemblage was more frequently detected in children between 3 and 10 years of age while AII was predominant in children under 2 years (Table 3). Although few BIV isolates were characterized, they were mostly identified in young children up to 2 years of age.

**Table 3:** Distribution of *G. duodenalis* sub-assemblages by gender and age

Frequency of sub-assemblages in children groups						
n (%)						
		AI	AII	BIII	BIV	*B
Gender	n	(n=15)	(n=47)	(n=4)	(n=9)	(n=5)
Female	43	10 (66.7)	24 (51.1)	1 (25)	5 (55.6)	3 (60)
Male	37	5 (33.3)	23 (48.9)	3 (75)	4 (44.4)	2 (40)
Age (years)						
0 - 2	40	2 (13.3)^a,b^	27 (57.4)^c^	1 (25.0)	7 (77.8)	3 (60)
3 - 6	31	8 (53.3)^a^	16 (34.0)^c^	3 (75.0)	2 (22,2)	2 (40)
7- 10	5	5 (33.3)^b^	0 (0.0)	0 (0)	0 (0.0)	0 (0.0)
11 - 14	4	0 (0)	4 (8.5)^c^	0 (0)	0 (0.0)	0 (0.0)

^a^ ^bc^ Equal letters indicate statistically significant differences (p<0.05, chi-squared test) in the frequency of sub-assemblages among the groups. * Non-subtyped assemblage B samples

## Discussion

Advances in molecular biology studies of *G. duodenalis* have shown that the parasite is a multispecies complex with little variation in their morphology but with a great genetic variability. This species is classified into eight distinct assemblages (A-H) but only A and B are regularly found in humans, although they can be detected in other domestic and wild animals ^8^^,^^10^^,^^36^^,^^37^.

Despite the high prevalence of giardiasis in Brazil, there are few studies on the genetic diversity of *G. duodenalis*. It is rare to find reports from the northeastern region of the country and there are no data of assemblage distribution in the state of Bahia.

In our study, we performed the molecular characterization of 110 isolates of *G. duodenalis* from children living in Salvador who were divided into two groups: children who attended day care centers and those who were seen in a public clinical laboratory. All the isolates were subjected to PCR to amplify *beta-giardin* and *gdh* gene fragments. Eighty (72.7%) samples were successfully amplified in at least one of the genes analyzed with slightly greater success in the amplification rate of *gdh* (67.2%) than in *beta-giardin* (53.6%). These genes are often used to detect and/or genotype *Giardia* isolates from fecal samples but differences in their amplifications have been reported ^27^^,^^30^ suggesting that the presence of divergences between the genomic sequences and primers used for PCR may result in the reduction or even lack of amplification ^37^^,^^38^.

Thirty (27.3%) of the isolates in this study did not amplify any of the genes tested. The negative PCR results could be explained by the presence of fecal DNA polymerase inhibitors, such as bilirubin, bile salts, hemoglobin, phenolic compounds, and complex polysaccharides, which are co-purified during the extraction of genomic DNA ^39^^,^^40^. These PCR inhibitors may vary in amount and specific characteristics depending on the diet of each individual.

In this study, we detected assemblages A (77.5%) and B (22.5%) with a significant predominance of the former, as found in Spain ^41^, Germany ^16^, Portugal ^15^, Uganda ^17^, Egypt ^42^, Syria ^18^, and Jamaica ^43^. These results contrast with those from Austria ^19^, Kenya ^20^, Libya ^21^, Canada ^22^, and Afghanistan ^44^ where a higher prevalence of assemblage B has been observed. In Latin America, assemblage B has been predominant in Colombia and Argentina, assemblage A in México ^45^^,^^46^ while no difference among these molecular groups was observed in Cuba ^47^.

In Brazil, due to the huge territorial dimension of the country, the prevalence of *G. duodenalis* assemblages varies between regions. Recent studies in day care children in Sao Paulo showed a predominance of assemblage B ^27^ while in pre-school children from a Rio de Janeiro slum, assemblage A was predominant ^26^. In Fortaleza ^28^, Minas Gerais ^29^, and Paraná ^48^, B molecular isolates were more frequent. However, in studies conducted in Amazonas ^49^, Rio de Janeiro ^30^, Sao Paulo ^50^, and Santa Catarina ^51^, assemblages A and B were found in similar proportions. It is important to note that the majority of studies in Brazil performed molecular characterization of less than 50 *G. duodenalis* isolates ^26^^,^^29^^,^^48^^,^^50^^-^^53^. In contrast, in our study, 80 isolates of *G. duodenalis* from different groups of children were analyzed.

Regarding our sampling, we found differences in *G. duodenalis* assemblage distribution between groups: In day care center 1, assemblage A was the most prevalent (83.9%; p<0,05) while no significant differences between A (54.2%) and B (45.8%) occurrence were observed in day care center 2. The dissemination of *G. duodenalis* cysts through person-to- person contact, common in day care centers, can promote the concentration of certain molecular isolates ^27^, which may justify the predominance of assemblage A in day care center 1. Additionally, the presence of more than one assemblage in day care center 2 reflects multiple sources of exposure possibly associated with the socioeconomic vulnerability of children seen at this center ^54^. Considering that the children from the public laboratory group came from locations in Salvador with no relationship among them, the predominance of assemblage A (92%) suggests a higher frequency of environmental dissemination of this molecular type in the city either through contaminated drinking water and/or food, greens, and other vegetables.

Regarding sub-assemblage characterization, AII was the most frequently detected in 58.8% of cases (47/80) followed by AI (18.8%), BIV (11.2%), and BIII (5.0%). Similarly, studies in other countries have reported a predominance of AII sub-assemblage in children ^18^^,^^41^^,^^55^^,^^56^ and also in some studies conducted in Brazil ^30^^,^^51^^,^^53^. However, our results contrast with studies conducted in Rio de Janeiro where most of the isolates were identified as AI ^25^ while in Paraná ^48^ and in a day care center in São Paulo ^27^ BIV predominated. The higher frequency of the AII sub-genotype in our study suggests that transmission of giardiasis occurs mainly through an anthroponotic route (direct or indirect) since this subtype is predominantly isolated from humans ^8^^,^^10^.

When we specifically analyzed the distribution of the sub-assemblages in the samples from the two day care centers, there was a higher occurrence of AII followed by BIII and BIV. The detection of these subtypes corroborates reports of the role of person-to-person transmission of giardiasis due to agglomeration of individuals in childcare centers since they are predominantly found in humans ^36^^,^^57^, a hypothesis also supported by the absence of the AI sub-assemblage, frequently found in domestic and livestock animals ^36^. On the other hand, AI sub-assemblage was detected in the majority of children (60.0%, 15/25) from the public clinical laboratory group. This molecular type is more frequently associated with infection in animals than in humans ^8^^,^^46^ suggesting that poor treatment of drinking water, contamination of water reservoirs with animal excreta, and/or contact with pets (dogs and cats) may be factors involved in the exposure to the parasite in this group.

The occurrence of mixed human infections involving different *G. duodenalis* molecular isolates has been reported in previous studies with rates varying from 2 to 21% and higher in developing countries ^7^^,^^18^^,^^20^^,^^33^^,^^45^^,^^58^. In our study, isolates with an RFLP pattern suggestive of mixed infections were not confirmed by *beta-giardin* gene sequencing. The occurrence of mixed infections by various *G. duodenalis* assemblages/sub-assemblages reflects the complex circulation of the parasite in the environment, the exposure of this population to multiple sources of infection, and the lack of cross-immunity between different molecular isolates ^20^. On the other hand, the occurrence of RFLP profiles suggestive of the concomitant presence of two or more genotypes in the same sample can also be attributed to the heterozygous allelic sequence of the target gene ^13^, as demonstrated by Morrison, *et al.*^59^, in the *G. duodenalis* genome project.

There were no significant differences in *G. duodenalis* sub-assemblage frequency by gender in this study, which agrees with previous reports ^18^^,^^60^, although another study found *G. duodenalis* molecular type B as the most frequent in females ^61^. Our results also showed that AII sub-assemblage frequency was significantly higher in the 0-2 year age group while the AI was higher in children 3-6 years of age. These results corroborate results from studies reporting a higher prevalence of AII genotype in younger children ^18^^,^^62^. The high infection rate of AII in younger children can be explained by sub-standard hygiene habits facilitating the transmission of this predominantly anthroponotic sub-assemblage. However, the higher frequency of AI infection in the 3-6 age range may be due to progressive contact with pets possibly facilitating the dissemination of this zoonotic isolate. Nevertheless, we cannot exclude the possibility of intestinal colonization by a new *G. duodenalis* molecular type due to active immunological memory against a previously eliminated isolate. In fact, studies conducted in Rio de Janeiro at different periods suggest the substitution of one *G. duodenalis* genetic isolate for another in the population ^25^^,^^30^.

In our study, 91.8% of the children infected with *G. duodenalis* did not have diarrhea or relevant gastrointestinal symptoms at the time of fecal analysis. Among the giardiasis cases analyzed, out of 9 children (8.2%) seen at a clinical analysis laboratory seven were symptomatic and had characterized while only two of them had diarrhea (both infected with AI sub-assemblage). The other five had other gastrointestinal symptoms (2 were infected with sub-assemblage AII, 2 with AI, and 1 with assemblage B). Given the limited number of symptomatic individuals in our study, it was not possible to evaluate associations between molecular isolates and symptoms. However, it is important to emphasize that asymptomatic children play a role as cyst disseminators in day care centers and in the environment.

Although most *G. duodenalis* carriers studied were asymptomatic (predominantly of assemblage A), it is important to highlight the similar distribution of A and B molecular groups in one of the day care centers suggesting that factors intrinsic to the host (age, nutritional status, immunological response, intestinal microbiota) are more relevant in triggering disease than the parasite molecular type involved.

This is one of the few studies of *G. duodenalis* genetic characterization undertaken in northeastern Brazil and the first one in the state of Bahia. The results show that although AII sub-assemblage predominated in the analyzed population suggesting that anthroponotic transmission is more common in our environment, there is a high molecular variability of *G. duodenalis* isolates, which evidences that zoonotic transmission routes can also be present. Apparently, in early childhood, there is a preferential susceptibility to AII *G. duodenalis* sub-assemblage, which changes to AI and possibly BIV in children over three years of age and is maybe related to the development of a subassemblage-specific immune response.

More studies analyzing different groups parasitized by *G. duodenalis* with a variety of clinical conditions are necessary for a better understanding of the molecular epidemiology of giardiasis.
